# Fabrication of (Co,Mn)_3_O_4_/rGO Composite for Lithium Ion Battery Anode by a One-Step Hydrothermal Process with H_2_O_2_ as Additive

**DOI:** 10.1371/journal.pone.0164657

**Published:** 2016-10-27

**Authors:** Zuohua Li, Yanhui Cui, Jun Chen, Lianlin Deng, Junwei Wu

**Affiliations:** 1 School of Civil and Environmental Engineering, Harbin Institute of Technology Shenzhen Graduate School, Shenzhen, China; 2 IoT Application Technology Center of NDT, Shenzhen Graduate School, Harbin Institute of Technology, Shenzhen, China; 3 School of Materials Science and Engineering, Harbin Institute of Technology Shenzhen Graduate School, Shenzhen Key Laboratory of Advanced Materials, Shenzhen, China; Beihang University, CHINA

## Abstract

Binary transition metal oxides have been regarded as one of the most promising candidates for high-performance electrodes in energy storage devices, since they can offer high electrochemical activity and high capacity. Rational designing nanosized metal oxide/carbon composite architectures has been proven to be an effective way to improve the electrochemical performance. In this work, the (Co,Mn)_3_O_4_ spinel was synthesized and anchored on reduced graphene oxide (rGO) nanosheets using a facile and single hydrothermal step with H_2_O_2_ as additive, no further additional calcination required. Analysis showed that this method gives a mixed spinel, i.e. (Co,Mn)_3_O_4_, having 2^+^ and 3^+^ Co and Mn ions in both the octahedral and tetrahedral sites of the spinel structure, with a nanocubic morphology roughly 20 nm in size. The nanocubes are bound onto the rGO nanosheet uniformly in a single hydrothermal process, then the as-prepared (Co,Mn)_3_O_4_/rGO composite was characterized as the anode materials for Li-ion battery (LIB). It can deliver 1130.6 mAh g^-1^ at current density of 100 mA g^-1^ with 98% of coulombic efficiency after 140 cycles. At 1000 mA g^-1^, the capacity can still maintain 750 mAh g^-1^, demonstrating excellent rate capabilities. Therefore, the one-step process is a facile and promising method to fabricate metal oxide/rGO composite materials for energy storage applications.

## Introduction

Recently more attention has been paid to environment friendly, sustainable, and efficient device for energy conversion and storage devices.[1, 2] Li-ion batteries (LIBs) with high specific capacity have received worldwide interest and an increase in research output.[3–5] However, the conventional graphite anode fails to meet the requirements for the fast-growing markets, for example in portable electronics and hybrid vehicles, due to limited power and energy density.[6, 7]

Transition metal oxides, especially M_3_O_4_ (M = Mn, Co or Fe) with a spinel structure, are one of the most promising anode materials for lithium ion batteries, due to availability of the elements and high capacity, which is more than twice that of traditional intercalation graphite anode. Metal oxide (M_3_O_4_) behaves as anode materials for LIB based on conversion (redox) reaction.[8] The mechanism can be described by M_3_O_4_ + 8Li^+^ + 8e^-^ → 4Li_2_O + 3M. Normally lithium oxide Li_2_O is stable and does not decompose to the metallic lithium, however, in the presence of nanosized transition metal (nano-M), the reaction can proceed as nano-M + Li_2_O ↔ 2Li^+^ + nano-MO, and thus the function as lithium ion battery anode can be achieved. Moreover, engineering the metal oxides at nanoscale offers further advantages, such as increased active surface areas, short ion diffusion pathways, and better accommodation of the reaction strains, which enhance the energy storage capacity or rate capabilities.

However, poor electronic conductivity and agglomeration of nanoparticles restricts the direct utilization of nanosized transition metal oxide and graphene nanosheets. Carbon nano fiber or other carbon source, which have better electronic conductivity,[9] can connect with each other to provide a fast electronic conduction path and offer a space framework for metal oxide nucleation to avoid agglomeration of the nanoparticles. As anode materials for lithium ion battery, the M_3_O_4_/C composites can also accommodate the volume expansion of active materials to achieve long term stable performances.[10] Yang et al.[11] reported that NiCo_2_O_4_/carbon nanocomposite was prepared using a hydrothermal method and achieved a high reversible capacity (958.4 mA h g^-1^ at a current of 40 mA g^-1^ after 50 cycles) as LIB anode. Composite materials of nano transition metal oxides with graphene had been reported extensively to have better electronic conductivity and electrochemical performances, such as nanomaterials of Co_3_O_4_,[12] Mn_3_O_4_,[13] CoMn_2_O_4_,[14,15] NiCo_2_O_4_,[11,16] ZnMn_2_O_4_[17] etc. However these reported synthesis methods typically involve a two-steps process, and a facile and simple method to synthesize M_3_O_4_/graphene composites with excellent lithium storage behavior is still a challenge, especially for binary oxides.

In this work, we demonstrate a one-step hydrothermal route to synthesize nanocube (Co,Mn)_3_O_4_ of about 20 nm in size, which are anchored on rGO. Unlike traditional processes, no post annealing is necessary to crystallize the nanocubes. The as-prepared (Co,Mn)_3_O_4_/rGO composite has been characterized as anode materials for a LIB. The obtained results show the (Co,Mn)_3_O_4_/rGO composite exhibits a high discharge capacity, long term cycling stability and an excellent rating capability. The optimum discharge capacity can reach 1130.6 mAh g^-1^ at 100 mA g^-1^ and a reversible capacity 939.1 mAh g^-1^ can be achieved after 140 cycles at 200 mA g^-1^, with capacity retention 98.8%.

## Experimental

### Synthesis of (Co,Mn)_3_O_4_/rGO composite

Graphene oxide (GO) was prepared by modified Hummers method.[18] In a typical procedure, 120 mg GO was dispersed in 30 ml deionized (DI) water by placing in an ultrasonic bath for 2 hours, 1.7 mmol Co(OAc)_2_ and 1 ml ammonium hydroxide (NH_3_·H_2_O), 3.5mmol Mn(OAc)_2_ were dissolved in 15 ml deionized (DI) water. The two solutions were then mixed and 2 ml H_2_O_2_ was added before the solution was transferred into 100 ml Teflon-lined stainless steel autoclave. The autoclave was kept at 200°C for 6 h before cooling down to room temperature. The precipitate was washed and then placed in a centrifuge with DI water, this procedure was repeated several times, until a pH 7 was obtained followed by freeze-drying to obtain a (Co,Mn)_3_O_4_/rGO composite. For comparison, rGO was attained to remove (Co,Mn)_3_O_4_ from (Co,Mn)_3_O_4_/rGO composite, which was conducted in 1M HCl solution with sonication, and followed by freeze-drying.

### Characterizations

The crystalline structure of the (Co,Mn)_3_O_4_/rGO was characterized by X-ray diffraction (XRD) by Rigaku D/max 2500PC system (Cu-Ka 40 kV, 200 mA) in the range between 10° and 80° with the scan rate of 8°/min. Field emission scanning electron microscopy (FE-SEM, Hitachi S4700, 15kV) and transmission electron microscope (TEM, JEOL JEM-2100F) were used to observe surface morphology and identify the phase composition of as-prepared (Co,Mn)_3_O_4_/rGO composite. Raman spectroscopy was obtained using a Renishaw inVia Raman spectrometer with 415 nm diode laser excitation to access the state of the GO. The X-ray photoelectron spectroscopy (XPS) analysis was performed on a VG ESCALAB MKⅡwith Al Kα (1486.6 eV) as X-ray source.

### Electrochemical measurements

For Li-ion battery measurement, the working electrodes was prepared by mixing the (Co,Mn)_3_O_4_/rGO composite material with acetylene black and polymer binder (polyvinylidene fluoride; PVDF) in a weight ratio of 70:20:10. The slurry was cast on a copper foil by a standard laboratory scale doctor-blade technique followed by drying in air for 1 h at 80°C, and then further dried in a vacuum oven (<0.1 mbar) at 120°C for 24 h. CR2032 coin-type cells were directly fabricated in an Ar-filled glove box for electrochemical tests, using an electrolyte of 1 mol L^-1^ LiPF_6_ in a mixture of ethylene carbonate (EC) and diethyl carbonate (DEC) (1: 1 in volume, battery grade, from Dongguan Shanshan Battery Materials Co. Ltd, China), with 10% vinylene carbonate (VC, 99% purity, from Aladdin Reagents Co., Ltd, Shanghai) as an additive. A metallic lithium foil served as the counter electrode. The cycle performance was performed on a Maccor 4300 battery test system (accuracy: 0.1%) at 25°C with the potential window from 0.01 to 3.0 V. Cyclic voltammetry (CV) tests were conducted on a CHI760D electrochemical workstation at a scan rate of 0.5 mV s^-1^.

## Results and Discussion

### Morphology and Structure Analysis

**Fig 1:**
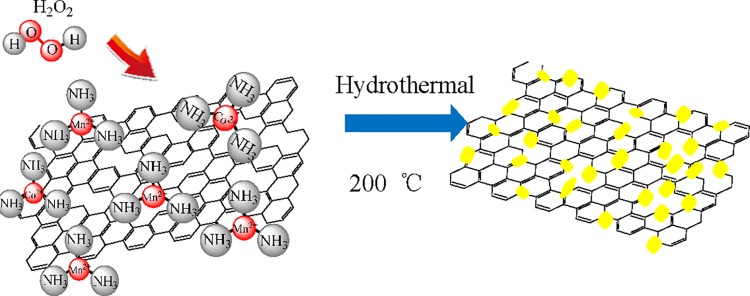
Schematic show of the synthesis of the (Co,Mn)_3_O_4_/rGO nanocubes.

A reaction process and the resulting novel anode material developed in this work are schematically illustrated in Fig 1.

In the first stage metal ions connect with ammonium hydroxide molecules to form metal amine complex, which are likely adsorbed on the surface of GO sheet with hydroxyl and carboxyl groups to form a GO + Co/Mn complex (left hand side of Fig 1). The introduction of H_2_O_2_ into the system oxidizes the transition metal ions (Mn^2+^, Co^2+^) partially to Mn^3+^, Co^3+^ and then in the hydrothermal process becomes well crystallized (Co,Mn)_3_O_4_ on rGO (right hand side of Fig 1). It is anticipated that the strongest adsorption sites result in (Co,Mn)_3_O_4_ and these sites have a even distribution on the GO. The reactions can be described by the following equations [19].

$$NH_{3}+H_{2}O\to {NH_{4}}^{+}+OH^{-}$$ (1)

$${\left[Co,Mn\right]}^{2+}+nNH_{3}\to {\left[\left(Co,Mn\right){\left(NH_{3}\right)}_{n}\right]}^{2+},n=1-2$$ (2)

$${\left[\left(Co,Mn\right){\left(NH_{3}\right)}_{n}\right]}^{2+}+2OH^{-}\to \left[\left(Co,Mn\right){\left(NH_{3}\right)}_{n}\right]{\left(OH\right)}_{2},n=1-2$$ (3)

$$3\left[\left(Co,Mn\right){\left(NH_{3}\right)}_{n}\right]{\left(OH\right)}_{2}+H_{2}O_{2}\to {\left(Co,Mn\right)}_{3}O_{4}+4H_{2}O+3nNH_{3},n=1-2$$ (4)

**Fig 2:**
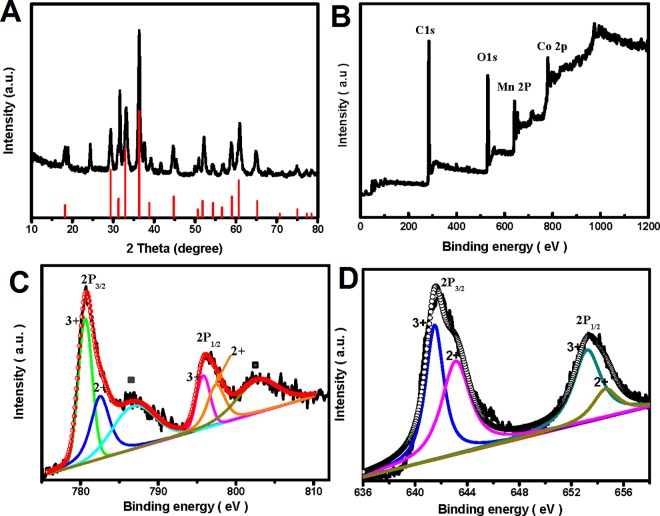
XRD and XPS characterizations (Co,Mn)_3_O_4_/rGO. (A) XRD patterns. (B) XPS survey spectrum. (C) High-resolution XPS spectra of Co 2p. (D) High-resolution XPS spectra of Mn 2p.

The as-synthesized (Co,Mn)_3_O_4_/rGO composite was first characterized by XRD to identify its crystallographic structure. As shown in Fig 2A, the main diffraction peaks of the composite material can be indexed to the spinel (Co,Mn)_3_O_4_ (JCPDF Card NO. 18–0408), and the sharp peaks prove that the (Co,Mn)_3_O_4_ spinel is well crystallized. A detailed analysis shows that the as-prepared product has two additional peaks around 24° and 31° matching well with MnCO_3_ (JCPDF Card No. 44–1472), suggesting a little amount of impurity. The accurate content of (Co,Mn)_3_O_4_/rGO composite shown in Figure A in S1 File reveals accordant element proportion with the raw material. The carbon state of the rGO was analysed by Raman spectroscopy. Clear D and G peaks are observed at 1350 cm^-1^ and 1590 cm^-1^ respectively (see Figure B in S1 File), which is consistent with standard spectrum seen for rGO.[20, 21] And I_D_/I_G_ of rGO is 1.08, lower than 1.16 of GO that is matching with the reference (Figure C Panel A in S1 File) [22]. The XRD profile comparison was shown in Figure C Panel B in S1 File, the XRD pattern of rGO also matches with the reference well [23], suggesting rGo was well prepared. A further comparison with other Co-Mn spinel oxides has been made as shown in Figure D and Table A in S1 File, and it is further verified that the product obtained here is a tetragonal mixed spinel with the composition (Co,Mn)_3_O_4._

A mixed spinel with the composition (Co,Mn)_3_O_4_ suggests that both tetragonal and octahedral sites in the spinel are occupied by both 2^+^ and 3^+^ oxidation states. Detailed elemental composition and oxidation state of as-synthesized product were characterized by XPS. The spectrum obtained in Fig 2B indicates the presence of Co, Mn, and O, as well as C from the rGO. In order to identify the oxidation state of Co and Mn, the XPS spectra in the Co 2p and Mn 2p region were analyzed and fitted (Fig 2C and 2D).[24] For Co (Fig 2C), based on the wide Co 2p_3/2_ and Co 2p_1/2_ peaks, it is reasonable to assume that Co^2+^ and Co^3+^ species co-exists.[24, 25] Whereas, two main peaks of Co 2p_3/2_ and 2p_1/2_ are fitted well with two spin-orbit doublets, characteristic of Co^2+^ and Co^3+^,[26–29] and two additional satellite peaks shown at 786.8 and 802.9 eV (indicarted by an inverted triangle) correspond to Co 2p_3/2_, Co 2p_1/2_ are respectively. The Mn 2p spectrum can be deconvolved into four peaks and assigned to the co-existence of Mn^2+^ and Mn^3+^ cations[30] as shown in Fig 2D. It is reasonable, therefore, to conclude that both of the Co and Mn elements have 2^+^ and 3^+^ valence states that agree well with the results of XRD test. And the solid-state redox couples Mn^3+^/Mn^2+^ and Co^3+^/Co^2+^ are present in the spinel structure, which may provide a notable electrochemical activity. Previously, CoMn_2_O_4_ microspheres[^31^^]^ and hierarchical porous CoMn(CoMn)_2_O_4_/rGO nanoplates[32] had been synethesized as electrode materials for supercapacitor applications. However, without any oxidant addition, such as H_2_O_2_, crystalline structure can only be obtained after calcinations at least 300°C.

**Fig 3:**
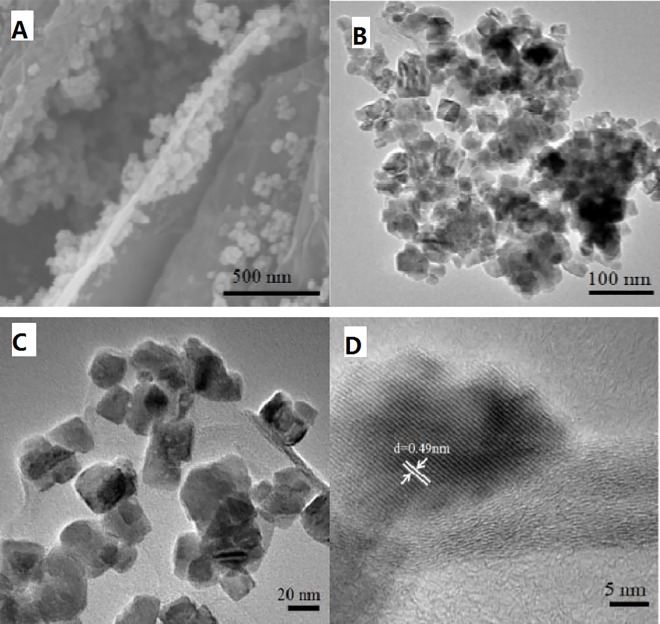
SEM and TEM images of (Co,Mn)_3_O_4_/rGO composite. (A) SEM images. (B) and (C) TEM images. (D) HRTEM images.

The morphology of the as-prepared composite was examined by SEM and TEM. Fig 3A shows a SEM image of the (Co,Mn)_3_O_4_/rGO composite, showing a large number of (Co,Mn)_3_O_4_ particles are anchored on the rGO nanosheets and Fig 3B shows that these nanoparticles distribute uniformly without any obvious agglomeration. Because the rGO sheets provide numerous nucleation sites, and as shown by the Fig 1 these are distributed on a scale of around 0.75 nm, but as seen in Fig 3C the distance between the cubic nanoparticles on the rGO sheet is around 30 nm, so there must be sites that are preferred for (Co,Mn)_3_O_4_ growth.

In addition, the higher magnification (scale bar = 20nm) in Fig 3C shows that the (Co,Mn)_3_O_4_ nanoparticles are ~20 nm with a cubic morphology. The HRTEM image shown in Fig 3D further reveals the (Co,Mn)_3_O_4_ nanocubes grown on rGO sheet have a lattice spacing of 0.49 nm, which corresponds well to the theoretical interplane spacing of spinel (Co,Mn)_3_O_4_ (111) planes.

### Li-Ion Batteries Performance

**Fig 4:**
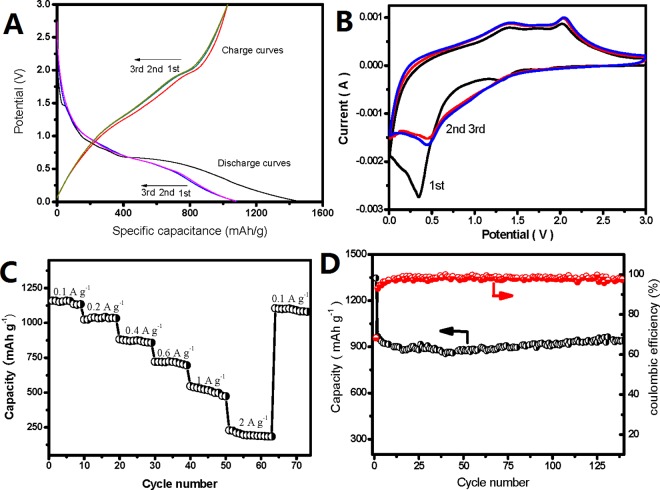
Electrochemical performances of (Co,Mn)_3_O_4_/rGO composite as lithium ion battery anode. (A) Charge-discharge voltage profiles of (Co,Mn)_3_O_4_/rGO composite for the first three cycles at a current density of 100 mA g^−1^. (B) CV curves for LIBs in the potential region of 0 ~ 3.0 V (vs. Li/Li^+^) at a scanning rate of 0.5 mV s^−1^. (C) A comparison of the specific capacity as a function of current density. (D) Cycling performance at current densities of 200 mA g^−1^ of (Co,Mn)_3_O_4_/rGO composite.

Coin-type cell was used to evaluate the lithium storage properties of (Co,Mn)_3_O_4_/rGO composite as lithium anode. Fig 4A shows the first three charge-discharge profiles of as-synthesized sample at a current density of 100 mA g^-1^ in the rage of 0.01~3.0 V (vs. Li/Li^+^).

The first discharge capacity is 1452 mAh g^-1^, and the first charge one is 1027 mAh g^-1^, showing an irreversible capacity loss of 400 mA h g^-1^, and a coulombic efficiency of 70.7%. The irreversible capacity loss arising during the first cycle is likely due to the incomplete decomposition of Li_2_O and the difficult dissolution of the SEI layer.[33] In the 2nd cycle, the discharge and charge capacity are 1082 mAh g^-1^ and 1026 mAh g^-1^ and in the 3rd cycle the discharge and charge capacity are 1076 mAh g^-1^ and 1028 mAh g^-1^, respectively. It is noted that the charge capacity are almost the same for the initial three cycles. The CV curves for LIBs at a scan rate of 0.5 mV s^-1^ in the potential range of 0.01~3V (vs. Li/Li^+^) are shown in Fig 4B. It is easily seen that the first cycle exhibits differs from the following 2 cycles which almost overlaps with each other, demonstrating that the electrode exhibit good stability and cyclability after 1^st^ cycle. In the first cycle, there is a broad peak at ~1.3 V and a sharp peak at ~0.3 V in the cathodic process, which corresponds to the reduction of Mn^3+^, Co^3+^ to Mn^2+^, Co^2+^ as well as Mn^2+^, Co^2+^ to metallic Mn, Co, respectively.[33] And it was also believed that the formation of SEI layer in the first discharge process will contribute to discharge capacity, which accounts for the larger discharge capacity and different CV shape with the next two cycles. Upon charge, two broad oxidation peaks are shown at 1.5 V and 2.0 V, which can be assigned to the oxidation of Mn and Co to Mn^2+^ and to Co^2+^, respectively. In the following two cycles, the reduction peak moves to about 0.5 V, which is different from the irreversible electrochemical reaction during the first discharge cycle.[34] On the basis of the cyclic voltammetry curves, the entire electrochemical process can be described as follows:

$${\left(Co,Mn\right)}_{3}O_{4}+8{Li}^{+}+8e^{-}\to 3Co+3Mn+4{Li}_{2}O$$ (5)

$$3Co+3Mn+6{Li}_{2}O↔3CoO+3MnO+12{Li}^{+}+12e^{-}$$ (6)

Previously, Qiang Shen and co-workers[35] found that MnCO_3_ spindle–GO composites and flower like MnCO_3_ could act as lithium ion battery anode materials. And the reaction mechanism can be expressed as:

$$MnCO_{3}+2Li\to {Li}_{2}CO_{3}+Mn$$ (7)

$${Li}_{2}CO_{3}+(4+0.5x)Li↔3{Li}_{2}O+0.5{Li}_{x}C_{2};(x=0,1or2)$$ (8)

However, when we make a comparison of the CV test (Fig 4B) in this work with those results of MnCO_3_ LIB anode[36–38], no extra peaks of MnCO_3_ was founded. Therefore, the MnCO_3_ impurity is neglectable to the electrode.

Fig 4C shows the rate capability of as-prepared (Co,Mn)_3_O_4_/rGO composite. The electrode delivers a specific capacity of 1131 mAh g^-1^ at 100 mA g^-1^, 951 mAh g^-1^ at 200mA g^-1^, 877 mAh g^-1^ at 400 mA g^-1^, 720 mAh g^-1^ at 600mA g^-1^, 546 mAh g^-1^ at 1000 mA g^-1^ and 212 mAh g^-1^ at 2000 mA g^-1^, respectively. When the current density turned back to 100 mA g^-1^, a specific capacity of 1105 mAh g^-1^ with a capacity retention of 97.8%, demonstrating excellent rate capabilities of the synthesized composite materials. Fig 4D shows cycling performances at the current density of 200 mA g^-1^. After 140 cycles, a reversible capacity 939 mAh g^-1^ can be obtained without any obvious degradation, with the capacity retention of 98.8%. In addition, the coulombic efficiency retains 98% after 140 cycles. The slight capacity increase after 50^th^ cycle is due to the improvement of Li^+^ accessibility and accommodation behavior in the electrode, the transformation of crystalline structure to an amorphous structure of (Co, Mn)_3_O_4_, and the formation of a gel-like surface film during the initial activation process[39–41].

Co-Mn oxides had been studied extensively as anode materials for lithium ion batteries. Here we made a comparison of the Co-Mn oxides in electrochemical performance and synthesis method, as summarized in Table 1. It is obvious that the (Co,Mn)_3_O_4_/rGO composites in this work exhibits outstanding cycling performance and highest reversible capacity. It is also noted that two steps process is the most popular way to prepare crystalline Co-Mn oxides in previous works, which prepared the precursor firstly followed by heat treatment to obtain the oxide. However in this work, we report a simple and facile route to achieve (Co,Mn)_3_O_4_/rGO hybrid without any following heat treatment. To the best of our knowledge, this is the first time to report one-step hydrothermal synthesize (Co,Mn)_3_O_4_/rGO composite as an anode for LIBs with outstanding performances. The excellent performance is attributed to synthetic effect of well distributed nanocubes and strong adherence on rGO, which guarantees rapid lithium conversion reaction and high electronic conductivity. Therefore, the one-step process is a facile and promising method to fabricate metal oxide/rGO composites for LIB applications. It can be further used to synthesize other transition metal oxide/rGO composites for various applications, such as electrochemical capacitor, and catalysts.

**Table 1:** Comparison of the Co-Mn oxides electrochemical performance between this work and the previous reports.

Complex oxide	Reversible capacity (mAh g^-1^)	Current density (mA g^-1^)	Capacity retention	Synthesis rout	Ref.
(Co,Mn)_3_O_4_/rGO composite	939.1/140^th^ cycle	200	98.8%	One step hydrothermal	This work
MnCo_2_O_4_ quasi-hollow microspheres	610/100^th^ cycle	400	89.7%	Solvothermal and annealing	[33]
Double-shelled CoMn_2_O_4_ hollow microcubes	624/50^th^ cycle	200	75.5%	Co-precipitation and annealing	[12]
CoMn_2_O_4_ powers	330/50^th^ cycle	80	70.2%	Co-precipitation and annealing	[38]
CoMn_2_O_4_ microsphere	894/65^th^ cycle	100	94.9%	Solvothermal and thermal decomposition	[13]
MnCo_2_O_4_ nanowire	895.8/50^th^ cycle	100	92.7%	hydrothermal and annealing	[42]
MnCo_2_O_4_ nanowire	450/30^th^ cycle	800	75.4%	Solvothermal and annealing	[43]
CNF@CoMn_2_O_4_ nanocable	870/150^th^ cycle	200	98%	Refluxing and annealing	[44]

## Conclusions

In summary, we firstly demonstrated an one-step route synthesis for binary transition metal oxide (Co,Mn)_3_O_4_ nanocubes on the surface of rGO. With the introduction of oxidant H_2_O_2_ in the synthesis process, the (Co,Mn)_3_O_4_/rGO composites can be obtained by a single hydrothermal process. As anode materials for LIBs, as-prepared composites delivered reversible capacity of 1100 mA h g^-1^ at a current density of 100 mA g^-1^. Also, excellent electrochemical stability is revealed with nearly 98.8% retention of initial capacity after 140 cycles at 200 mA g^-1^. We believe such good performance results from nano sized and uniform growth of cube (Co,Mn)_3_O_4_ particles on rGO, which would shorten the transfer distance of ions and increase the active contact area with electrolyte. Therefore, the one-step process is a simple and promising method to fabricate high performance binary metal oxide/rGO composites for LIB applications. Furthermore, the one-step route can be expanded to synthesize other binary transition oxides for various applications.

## Supporting Information

S1 File Figure A. The EDS spectrum and results of (Co, Mn)_3_O_4_/rGO composite; Figure B. Raman spectra of the (Co,Mn)_3_O_4_/rGO composite; Figure C. The Raman spectra and XRD patterns of GO and rGO. (A) The Raman spectra. (B) XRD patterns of GO and rGO. The rGO is obtained by removing the metal oxide of (Co, Mn)_3_O_4_/rGO; Figure D. (Co,Mn)_3_O_4_ XRD spectra comparison. (A) (Co,Mn)_3_O_4_ with CoMn_2_O_4_, MnCo_2_O_4_. (B) (Co,Mn)_3_O_4_ with Co_3_O_4_, Mn_3_O_4_; Table A. Detailed information of Co-Mn oxides.
(DOCX)
