# Supplementary material for: Fabrication of (Co,Mn)3O4/rGO Composite for Lithium Ion Battery Anode by a One-Step Hydrothermal Process with H2O2 as Additive
Source: PLoS One. 2016 Oct 27;11(10):e0164657. doi: 10.1371/journal.pone.0164657 (PMC5082892; doi:10.1371/journal.pone.0164657)
Supplement: S1 File — Figure A. The EDS spectrum and results of (Co, Mn)3O4/rGO composite; Figure B. Raman spectra of the (Co,Mn)3O4/rGO composite; Figure C. The Raman spectra and XRD patterns of GO and rGO. (A) The Raman spectra. (B) XRD patterns of GO and rGO. The rGO is obtained by removing the metal oxide of (Co, Mn)3O4/rGO; Figure D. (Co,Mn)3O4 XRD spectra comparison. (A) (Co,Mn)3O4 with CoMn2O4, MnCo2O4. (B) (Co,Mn)3O4 with Co3O4, Mn3O4; Table A. Detailed information of Co-Mn oxides. (DOCX) [file pone.0164657.s001.docx]

**Electronic Supplementary Information**

Fabrication of (Co,Mn)_3_O_4_/rGO composite for lithium ion battery anode by a One-step hydrothermal process with H_2_O_2_ as additive

Zuohua Li^1,3^, Yanhui Cui^2^, Jun Chen^2^, Lianlin Deng^2^, Junwei Wu^2,3^*

1. School of Civil and Environmental Engineering, Harbin Institute of Technology Shenzhen Graduate School, Shenzhen 518055, China

2. School of Materials Science and Engineering, Harbin Institute of Technology Shenzhen Graduate School, Shenzhen Key Laboratory of Advanced Materials, Shenzhen 518055, China.

3. IoT Application Technology Center of NDT, Shenzhen Graduate School, Harbin Institute of Technology, Shenzhen 518055, China

Corresponding author: [junwei.wu@hitsz.edu.cn](mailto:junwei.wu@hitsz.edu.cn).


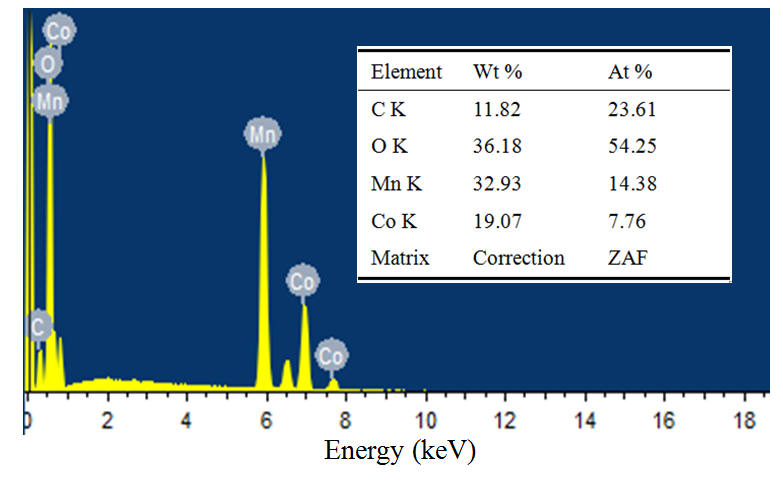


Fig A. The EDS spectrum and results of (Co, Mn)_3_O_4_/rGO composite.

Fig B. Raman spectra of the (Co,Mn)_3_O_4_/rGO composite.

Fig C. The Raman spectra and XRD patterns of GO and rGO

(A) The Raman spectra. (B) XRD patterns of GO and rGO. The rGO is obtained by removing the metal oxide of (Co, Mn)_3_O_4_/rGO.

Fig D. (Co,Mn)_3_O_4_ XRD spectra comparison

(A) (Co,Mn)_3_O_4_ with CoMn_2_O_4_, MnCo_2_O_4_. (B) (Co,Mn)_3_O_4_ with Co_3_O_4_, Mn_3_O_4_.

Here, we made a detailed comparison among CoMn_2_O_4_ (JCFD card 77-0471), MnCo_2_O_4_ (JCFD card 23-1237), Co_3_O_4_ (JCFD card 43-1003), Mn_3_O_4_ (JCFD card 24-0734) and (Co,Mn)_3_O_4_ (JCPDF card 14-0408), as shown in Fig.S4. By comparing with CoMn_2_O_4_ and MnCo_2_O_4_ in Fig.S4a, some peaks show a slight shift. Therefore, (Co,Mn)_3_O_4_ was used here. In addition, a comparison with Co_3_O_4_, Mn_3_O_4_ are made in Fig.S4b. The peaks shift even more than the mixed oxides. Table S1 exhibit the detailed XRD information of these Co-Mn oxides.

Table A. Detailed information of Co-Mn oxides.

| Co-Mn oxide | Structure | 2θ of the strongest peak | [Crystal](C:/Users/Administrator/AppData/Local/Yodao/DeskDict/frame/20151202151418/javascript:void(0);) [face](C:/Users/Administrator/AppData/Local/Yodao/DeskDict/frame/20151202151418/javascript:void(0);) | PDF card No. | RIR |
| --- | --- | --- | --- | --- | --- |
| as prepared |  | 36.341 |  |  |  |
| (CoMn)(CoMn)_2_O_4_ | Tetragonal | 36.357 | (311) | 14-0408 |  |
| Mn_3_O_4_ | Tetragonal | 36.085 | (211) | 24-0734 | 1.5 |
| CoMn_2_O_4_ | Tetragonal | 36.071 | (211) | 77-0471 | 2.89 |
| MnCo_2_O_4_ | Cubic | 35.995 | (311) | 23-1237 | 1.3 |
| Co_3_O_4_ | Cubic | 36.845 | (311) | 43-1003 | 4.3 |
